# Tracking Cryptosporidium parvum by sequence analysis of small double-stranded RNA.

**DOI:** 10.3201/eid0701.010121

**Published:** 2001

**Authors:** L Xiao, J Limor, C Bern, A A Lal, Epidemic Working Group

**Affiliations:** Division of Parasitic Diseases, Centers for Disease Control and Prevention, Mail Stop F12, Atlanta, GA 30333, USA. lax0@cdc.gov

## Abstract

We sequenced a 173-nucleotide fragment of the small double-stranded viruslike RNA of Cryptosporidium parvum isolates from 23 calves and 38 humans. Sequence diversity was detected at 17 sites. Isolates from the same outbreak had identical double-stranded RNA sequences, suggesting that this technique may be useful for tracking Cryptosporidium infection sources.


### Tracking *Cryptosporidium parvum* by Sequence Analysis of Small Double-Stranded RNA

**Lihua Xiao, Josef Limor, Caryn Bern, and Altaf A. Lal**
Centers for Disease Control and Prevention, Atlanta, Georgia, USA

We sequenced a 173-nucleotide fragment of the small double-stranded viruslike RNA of *Cryptosporidium parvum* isolates from 23 calves and 38 humans. Sequence diversity was detected at 17 sites. Isolates from the same outbreak had identical double-stranded RNA sequences, suggesting that this technique may be useful for tracking *Cryptosporidium* infection sources.

*Cryptosporidium* parasites cause infection in humans and other vertebrates. Two genotypes of *Cryptosporidium parvum* are responsible for most cases of human infection; the human genotype (genotype 1 or anthroponotic genotype) is found almost exclusively in humans, whereas the bovine genotype (genotype 2 or zoonotic genotype) is found in both ruminants and humans (1-4). In addition to zoonotic and person-to-person transmission, both genotypes of *C. parvum* have caused waterborne and foodborne outbreaks. Current genotyping tools permit only differentiation of *Cryptosporidium* parasites at the genotype level, which limits ability to track infection and contamination sources in outbreaks.

Two double-stranded (ds) extrachromosomal viruslike RNAs have recently been identified in *C. parvum* (5). Both ds-RNAs have been found in all *C. parvum* oocysts examined. Sequence analysis of both the small and large ds-RNAs from seven *C. parvum* human genotype isolates and five bovine genotype isolates showed distinct ds-RNA sequences in isolates from the same genotype (6), indicating that ds-RNA has potential as a subgenotyping tool for *Cryptosporidium*. We report sequence diversity in the small ds-RNA of *C. parvum* human and bovine genotype isolates and discuss the usefulness of this technique for laboratory investigations and for tracking the source of cryptosporidiosis outbreaks.

Address for correspondence: Lihua Xiao, Division of Parasitic Diseases, Centers for Disease Control and Prevention, Mail Stop F12, Atlanta, GA 30333, USA; fax: 770-488-4454; e-mail: lax0@cdc.gov.

### The Study

We sequenced the small ds-RNA of 61 *C. parvum* isolates (23 isolates from cattle and 38 from humans) (Table). Eighteen of the 38 human isolates were from two foodborne outbreaks (Spokane, Washington, 1997; and Washington, D.C., 1998) and one waterborne outbreak (Minnesota, 1997) with well-defined infection sources (7-9). These isolates had been genotyped by polymerase chain reaction (PCR)-restriction fragment length polymorphism analysis of the SSU rRNA and TRAP-C2 genes (10,11). All bovine isolates and the human isolates from the Minnesota outbreak were of the *C. parvum* bovine genotype, and the other human isolates were of the *C. parvum* human genotype (Table). Total nucleic acid was extracted from purified oocysts or oocyst-containing fecal materials by the phenol-chloroform method (11) and stored at -20°C before molecular analysis.

A 173-nucleotide fragment of small ds-RNA was amplified by reverse-transcription (RT)-PCR with the GeneAmp RNA PCR Core kit (PE Applied Biosystems, Foster City, CA), according to the manufacturer's protocol. Random primers were used, and the nucleic acid was preheated at 65°C for 30 min. An aliquot (2 µL) of the RT mixture was used for PCR. The primers used were 5'-TGCAGTTTACTATCCAGTGG-3' and 5'-GCAGAAGGGTTCTATGATTC-3', and the PCR conditions were those described by Khramtsov et al. (5). PCR products were sequenced on an ABI 377 Automated Sequencer (Perkin Elmer, Foster City, CA). Sequence accuracy was confirmed by two-directional sequencing and sequencing of a second RT-PCR product. Nucleotide sequences

Table. *Cryptosporidium parvum* isolates used in this study*

| Isolate | Host | Source | Genotype | ds-RNA sequence type |
|---|---|---|---|---|
| 6 | Calf | Ohio, 1996[a] | Bovine | B |
| 7 | Calf | Ohio, 1996 | Bovine | M |
| 11 | Calf | Ohio, 1996 | Bovine | G |
| 16 | Calf | Ohio, 1996 | Bovine | B |
| 28 | Calf | Ohio, 1996 | Bovine | G |
| 45 | Calf | Ohio, 1996 | Bovine | D |
| 46 | Calf | Ohio, 1996 | Bovine | G |
| 49 | Calf | Ohio, 1996 | Bovine | G |
| 50 | Calf | Ohio, 1996 | Bovine | D |
| 51 | Calf | Ohio, 1996 | Bovine | G |
| 53 | Calf | Ohio, 1996 | Bovine | B |
| 57 | Calf | Ohio, 1996 | Bovine | D |
| 3 | Calf | Oklahoma, 1996 | Bovine | D |
| 29 | Calf | Oklahoma, 1996 | Bovine | D |
| 89 | Calf | Pennsylvania, 1997 | Bovine | D |
| 21 | Calf | Idaho, 1996 | Bovine | F |
| 37 | Calf | Utah, 1996 | Bovine | M |
| 1346 | Calf | California, 1999 | Bovine | G |
| 1347 | Calf | California, 1999 | Bovine | G |
| 43 | Human via calf | Maryland, 1996[b] | Bovine | A |
| Beltsville | Calf | Maryland, 1996 | Bovine | H |
| AUCP | Calf | Alabama, 1996 | Bovine | A |
| KSU-1 | Calf | Kansas, 1996 | Bovine | A |
| 1676 | Human | Peru, 1995[c] | Human | J |
| 1677 | Human | Peru, 1996 | Human | J |
| 1683 | Human | Peru, 1997 | Human | I |
| 1684 | Human | Peru, 1997 | Human | I |
| 1685 | Human | Peru, 1997 | Human | I |
| 1902 | Human | Kenya, 1999[d] | Human | Q |
| 1904 | Human | Kenya, 1999 | Human | Q |
| 1905 | Human | Kenya, 1999 | Human | R |
| 1911 | Human | Kenya, 1999 | Human | R |
| 1927 | Human | Kenya, 1999 | Human | R |
| 1935 | Human | Kenya, 1999 | Human | R |
| HGA5 | Human | Georgia, 1995 | Human | N |
| HNO3 | Human | New Orleans, 1997[e] | Human | N |
| HNO6 | Human | New Orleans, 1997 | Human | E |
| HNO23 | Human | New Orleans, 1998 | Human | P |
| HNO27 | Human | New Orleans, 1998 | Human | N |
| HNO30 | Human | New Orleans, 1998 | Human | N |
| HNO32 | Human | New Orleans, 1998 | Human | N |
| HNO35 | Human | New Orleans, 1998 | Human | O |
| HNO52 | Human | New Orleans, 1999 | Human | N |
| HMOB1 | Human | Minnesota outbreak, 1997 | Bovine | C |
| HMOB3 | Human | Minnesota outbreak, 1997 | Bovine | C |
| HMOB4 | Human | Minnesota outbreak, 1997 | Bovine | C |
| HMOB5 | Human | Minnesota outbreak, 1997 | Bovine | C |
| HWA1 | Human | Spokane outbreak, 1997 | Human | L |
| HWA3 | Human | Spokane outbreak, 1997 | Human | L |
| HWA4 | Human | Spokane outbreak, 1997 | Human | L |
| HWA5 | Human | Spokane outbreak, 1997 | Human | L |
| HWA6 | Human | Spokane outbreak, 1997 | Human | L |
| HDC1 | Human | Washington, DC, outbreak, 1998 | Human | K |
| HDC2 | Human | Washington, DC, outbreak, 1998 | Human | K |
| HDC6 | Human | Washington, DC, outbreak, 1998 | Human | K |
| HDC7 | Human | Washington, DC, outbreak, 1998 | Human | K |
| HDC14 | Human | Washington, DC, outbreak, 1998 | Human | K |
| HDC16 | Human | Washington, DC, outbreak, 1998 | Human | K |
| HDC23 | Human | Washington, DC, outbreak, 1998 | Human | K |
| HDC25 | Human | Washington, DC, outbreak, 1998 | Human | K |

[a]The Ohio bovine samples were collected from four dairy farms in central Ohio over a 12-month period.
[b]The dates for laboratory isolates (Beltsville, KSU-1, and AUCP) were dates that oocyst passages were harvested for DNA extraction.
[c]Peruvian samples 1683, 1684, and 1685 were taken from the same patient on different days.
[d]Kenyan samples were collected from patients visiting two hospitals in Nairobi.
[e]The New Orleans samples were from HIV-positive patients.
ds = double-stranded from all isolates were aligned, and the relationship between isolates was assessed by unweighted pair group method with arithmetic means, by using the Wisconsin Package Version 9.0 (Genetics Computer Group, Madison, WI).

Eighteen distinct nucleotide sequences were obtained from the 61 isolates, dividing the 23 isolates of *C. parvum* bovine genotype into 8 subgenotypes (A,B,C,D,F,G,H, and M) and the 38 isolates of the human genotype into 10 subgenotypes (E,I,J,K,L,N,O,P,Q, and R). Subgenotype A sequence was identical to that obtained from the laboratory isolate KSU-1, whereas others showed 1- to 13-nucleotide differences from KSU-1 at 17 positions over the 173-nucleotide fragment of the small ds-RNA. Isolates of the *C. parvum* bovine genotype generally had more similarity in small ds-RNA sequences to KSU-1 (subgenotype A) than those of the *C. parvum* human genotype. However, no nucleotide changes indicative of the genotypes (bovine or human) were present in the 173-nucleotide fragment (Figure 1).

Phylogenetic analysis was inconsistent in separating isolates of the *C. parvum* bovine genotype from those of the human genotype (Figure 2). However, isolates from the same outbreak clustered together: all isolates from the Washington, D.C., outbreak (subgenotype K); the Spokane outbreak (subgenotype L); and the Minnesota outbreak (subgenotype C) had identical ds-RNA sequences (Table, Figure 2). Similarly, a subgenotype (such as subgenotypes B, N, and R) was sometimes present in several isolates from the same geographic location. Some subgenotypes (for example, D and G) had broad

```

KSU-1    TGCAGTTTACTATCCAGTGGATTTGAAATTTGTCACTGACTTATCTTCAGATCTTTCGAATACAGCTGACGGATTAGGCCAGGCTTGGTA 90

A        ..........................................................................................

B        ...............................................................G..........................

C        ...........................................................................G..............

D        ..........................................................................................

E        ..........................................................................................

F        ...............................................................G..........................

G        ...............................................................G..........................

H        ..........................................................................................

I        ...............................................................G..........................

J        ................................................G..............G..........................

K        ...............................................................G..........................

L        ..........................................................................................

M        ...............................................................G..........................

N        ...............................................................G..........................

O        ..........................................................................................

P        ................................................G......G.........................A........

Q        ................................................G......G.......G.................A........

R        ................................................G......G.........................A........

KSU-1    TAAAATTTCACAAGTCGCAGTAGAGCATATAATCCTAACGGCATTGAAGATAAATTATGTCTTGAATCATAGAACCCTTCTGC 173
A        ...................................................................................
B        ...................................................................................
C        .......................................A...........................................
D        ....................................G..A...........................................
E        ................A....C.................A...........................................
F        ...........................C...........A...........................................
G        ....................................G..A...........................................
H        ..................................T.G..A...........................................
I        .....................T.................A...........................................
J        ..................................T....A...........................................
K        ..................................T....A.................C.........................
L        C....................T............T....A...........................................
M        C.................................T.G..A.................C.........................
N        ..................................T.G..A.................C.........................
O        ..................................T.G..A.................C.........................
P        ..............CAA.G.....A..A........T..A...........................................
Q        C.............C.A.G.....A..A........C..A...........................................
R        C.............CTA.G..G..A..A........C..A...........................................
```

Figure 1. Sequence diversity in the 173-nucleotide fragment of double-stranded RNA of *Cryptosporidium parvum*. Dots denote nucleotides identical to the KSU-1 isolate of the *C. parvum* bovine genotype. Representative sequences for each subgenotype were deposited in GenBank under accession numbers AF266262 to AF266277.

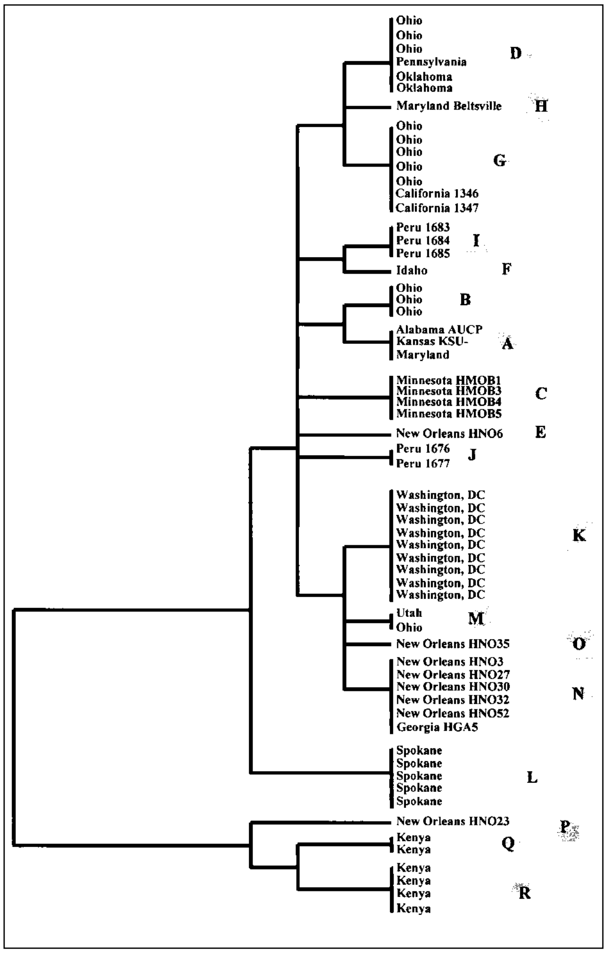

Figure 2. Genetic relationships of various subgenotypes of *Cryptosporidium parvum* human and bovine genotypes inferred by the unweighted pair group method with arithmetic means analysis of the small double-stranded RNA.

geographic distribution, and isolates from a given geographic area (such as those from calves in Ohio and humans in New Orleans) frequently had several subgenotypes.

### Conclusions

Subgenotyping tools are needed for studies of the molecular epidemiology of cryptosporidiosis. Such tools would facilitate laboratory characterization of cryptosporidiosis outbreaks and identification of contamination and infection sources. Analysis of the variations in subgenotype occurrence may also shed light on the transmission dynamics of *Cryptosporidium* parasites in different geographic areas and epidemiologic settings. The extensive intragenotypic heterogeneity in the small ds-RNA sequence exhibited by isolates of the *C. parvum* bovine and human genotypes indicates that ds-RNA has potential as a high-resolution tool for subgenotyping *Cryptosporidium* parasites.

Our analysis of outbreak specimens illustrates the potential utility of subgenotyping tools for epidemiologic investigations. The waterborne outbreak in Minnesota affected children who played around a water fountain in a zoo (7). All four isolates had the same subgenotype (C), confirming that the children's infections came from the same source. In the foodborne outbreak in Spokane, which affected attendees at a holiday party (8), all five isolates analyzed had the subgenotype L sequence of the *C. parvum* human genotype, supporting the epidemiologic conclusion of a single source. The outbreak in Washington, D.C., was attributed to contamination of food by a food-handler who had symptomatic cryptosporidiosis in the week before the outbreak (9). As in the other outbreaks, all eight isolates were of the subgenotype K of the *C. parvum* human genotype, again confirming a common source. Analysis of a sample (HDC14) from the food-handler demonstrated a small ds-RNA sequence identical to those from the outbreak cases, providing further evidence that the food-handler was the likely source of the oocysts that caused the outbreak.

The presence of multiple subgenotypes at the same geographic location, the wide distribution of certain subgenotypes, and the apparent geographic segregation of some subgenotypes seen in this preliminary study highlight the complexity of cryptosporidiosis epidemiology. The two subgenotypes of *C. parvum* in Kenya were quite divergent from isolates from other areas, which suggests localized transmission cycles. This hypothesis is further supported by the predominance of one subgenotype (N) in New Orleans AIDS patients. However, the presence of four subgenotypes (B,D,G, and M) of the *C. parvum* bovine genotype in calves in central Ohio suggests that multiple *C. parvum* parasites of the same genotype can circulate simultaneously in a region. Both phenomena may occur in any given locality, leading to the pattern seen in eight specimens from AIDS patients in New Orleans, where five specimens were subgenotype N and the other three specimens were of three different subgenotypes. Analysis of more isolates from diverse locations is needed for a firm extrapolation of data.

A disadvantage of the ds-RNA subgenotyping tool is lack of specificity at the genotype level. Perhaps as a result of the use of a short fragment as the target, this technique does not distinguish the two genotypes of *C. parvum* and must therefore be used in combination with routine genotyping tools. Initial attempts targeted longer fragments of the large and small ds-RNAs. However, the RT-PCR that targeted longer fragments in amplifying samples of the *C. parvum* human genotype was much less efficient, probably because of sequence diversity at the primer regions and lower efficiency of reverse transcription of longer fragments. A recent sequence analysis by Khramtsov et al. of five isolates of the *C. parvum* bovine genotype and seven isolates of the human genotype consistently separated the two genotypes in both the large and small ds-RNAs (6). It remains to be determined whether these primers and others can be developed for sensitive genotyping and subgenotyping *Cryptosporidium* parasites.

### Acknowledgments

We thank Anne Moore, Barbara Herwaldt, Michael Arrowood, Bruce Anderson, William Shulaw, A. Morse, J. Inungu, Ronald Fayer, Robert H. Gilman, Lilia Cabrera, William Checkley, and Wangeci Ndiritu for providing *Cryptosporidium* isolates.

This work was supported in part by funds from the Food Safety Initiative, Centers for Disease Control and Prevention.

Dr. Xiao is a senior staff fellow in the Division of Parasitic Diseases, CDC. His research interests focus on the molecular epidemiology of enteric protozoa and malaria vaccine development.

### References

1. Morgan UM, Constantine CC, O'Donoghue P, Meloni BP, O'Brien PA, Thompson RCA. Molecular characterization of *Cryptosporidium* isolates from humans and other animals using random amplified ploymorphic DNA analysis. Am J Trop Med Hyg 1995;52:559-64.
2. Bonnin A, Fourmaux MN, Dubremetz JF, Nelson RG, Gobet P, Harly G, et al. Genotyping human and bovine isolates of *Cryptosporidium parvum* by polymerase chain reaction-restriction fragment length polymorphism analysis of a repetitive DNA sequence. FEMS Microbiol Lett 1996;137:207-11.
3. Peng MP, Xiao L, Freeman AR, Arrowood MJ, Escalante A, Weltman AC, et al. Genetic polymorphism among *Cryptosporidium parvum* isolates supporting two distinct transmission cycle. Emerg Infect Dis 1997;3:1-9.
4. McLauchlin J, Pedraza-Diaz S, Amar-Hoetzeneder C, Nichols GL. Genetic characterization of *Cryptosporidium* strains from 218 patients with diarrhea diagnosed as having sporadic cryptosporidiosis. J Clin Microbiol 1999;37:3153-8.
5. Khramtsov NV, Woods KM, Nesterenko MV, Dykstra CC, Upton SJ. Virus-like, double-stranded RNAs in the parasitic protozoan *Cryptosporidium* parvum. Mol Microbiol 1997;26:289-300.
6. Khramtsov NV, Chung PA, Dykstra CC, Griffiths JK, Morgan UM, Arrowood MJ, et al. Presence of double-stranded RNAs in human and calf isolates of *Cryptosporidium* parvum. J Parasitol 2000;86:275-82.
7. Centers for Disease Control and Prevention. Outbreak of cryptosporidiosis associated with a water sprinkler fountain–Minnesota, 1997. MMWR Morb Mortal Wkly Rep 1998;47:856-9.
8. Centers for Disease Control and Prevention. Foodborne outbreak of cryptosporidiosis–Spokane, Washington, 1997. MMWR Morb Mortal Wkly Rep 1998;47:565-6.
9. Quiroz ES, Bern C, MacArthur JR, Xiao L, Fletcher M, Arrowood MJ, et al. An outbreak of cryptosporidiosis linked to a foodhandler. J Infect Dis 2000;181:695-700.
10. Sulaiman IM, Xiao L, Yang C, Moore A, Beard CB, Arrowood MJ, et al. Differentiating human from animal isolates of *Cryptosporidium parvum*. Emerg Infect Dis 1998;4:681-5.
11. Xiao L, Morgan U, Limor J, Escalante A, Arrowood M, Shulaw W, et al. Genetic diversity within *Cryptosporidium parvum* and related species of *Cryptosporidium*. Appl Environ Microbiol 1999;65:3386-91.

## References

[PDF_00315] Bonnin A., Fourmaux M. N., Dubremetz J. F., Nelson R. G., Gobet P., Harly G., Buisson M., Puygauthier-Toubas D., Gabriel-Pospisil G., Naciri M. (1996). Genotyping human and bovine isolates of Cryptosporidium parvum by polymerase chain reaction-restriction fragment length polymorphism analysis of a repetitive DNA sequence.. FEMS Microbiol Lett.

[PDF_00339] Centers for Disease Control and Prevention (CDC) (1998). Outbreak of cryptosporidiosis associated with a water sprinkler fountain--Minnesota, 1997.. MMWR Morb Mortal Wkly Rep.

[PDF_00335] Khramtsov N. V., Chung P. A., Dykstra C. C., Griffiths J. K., Morgan U. M., Arrowood M. J., Upton S. J. (2000). Presence of double-stranded RNAs in human and calf isolates of Cryptosporidium parvum.. J Parasitol.

[PDF_00331] Khramtsov N. V., Woods K. M., Nesterenko M. V., Dykstra C. C., Upton S. J. (1997). Virus-like, double-stranded RNAs in the parasitic protozoan Cryptosporidium parvum.. Mol Microbiol.

[PDF_00326] McLauchlin J., Pedraza-Díaz S., Amar-Hoetzeneder C., Nichols G. L. (1999). Genetic characterization of Cryptosporidium strains from 218 patients with diarrhea diagnosed as having sporadic cryptosporidiosis.. J Clin Microbiol.

[PDF_00310] Morgan U. M., Constantine C. C., O'Donoghue P., Meloni B. P., O'Brien P. A., Thompson R. C. (1995). Molecular characterization of Cryptosporidium isolates from humans and other animals using random amplified polymorphic DNA analysis.. Am J Trop Med Hyg.

[PDF_00346] Quiroz E. S., Bern C., MacArthur J. R., Xiao L., Fletcher M., Arrowood M. J., Shay D. K., Levy M. E., Glass R. I., Lal A. (2000). An outbreak of cryptosporidiosis linked to a foodhandler.. J Infect Dis.

[PDF_00349] Sulaiman I. M., Xiao L., Yang C., Escalante L., Moore A., Beard C. B., Arrowood M. J., Lal A. A. (1998). Differentiating human from animal isolates of Cryptosporidium parvum.. Emerg Infect Dis.

[PDF_00353] Xiao L., Morgan U. M., Limor J., Escalante A., Arrowood M., Shulaw W., Thompson R. C., Fayer R., Lal A. A. (1999). Genetic diversity within Cryptosporidium parvum and related Cryptosporidium species.. Appl Environ Microbiol.

